# Genome Sequence of *Ophidiomyces ophiodiicola*, an Emerging Fungal Pathogen of Snakes

**DOI:** 10.1128/genomeA.00677-17

**Published:** 2017-07-27

**Authors:** Mana Ohkura, Robert R. Fitak, Jennifer H. Wisecaver, Dan DeBlasio, Faheem Niazi, Michael Egholm, Steven D. Rounsley, Chinnappa D. Kodira, Marc J. Orbach

**Affiliations:** a School of Plant Sciences, University of Arizona, Tucson, Arizona, USA; b 454 Life Sciences, a Roche Company, Branford, Connecticut, USA

## Abstract

*Ophidiomyces ophiodiicola*, which belongs to the order *Onygenales*, is an emerging fungal pathogen of snakes in the United States. This study reports the 21.9-Mb genome sequence of an isolate of this reptilian pathogen obtained from a black racer snake in Pennsylvania.

## GENOME ANNOUNCEMENT

*Ophidiomyces ophiodiicola* is a keratinophilic fungal pathogen of the order *Onygenales* and is an emerging threat to wild and captive snakes in the United States (1). Isolates have also been reported in Germany, England, and Australia (2, 3). In the United States, *O. ophiodiicola* has been described on several snake species in the Northeast, the Southeast, and the Midwest; it is often characterized by skin lesions around the head but is also known to produce invasive infections. This pathogen belongs to the family *Onygenaceae*, which includes the saprotroph *Uncinocarpus reesei* and the human pathogens belonging to the genus *Coccidioides*. To develop an understanding of how pathogenicity on snakes evolved in this fungus, we sequenced and assembled its genome.

The isolate of *O. ophiodiicola* used for sequencing was recovered from a skeletal lesion of a captive black racer (*Coluber constrictor*) at the Pennsylvania State Animal Diagnostic Laboratory. Taxonomic analysis of this fungus was performed (4), and the isolate was deposited in the University of Arizona Mycological Herbarium (MYCO-ARIZ AN0400001). Genomic DNA of the isolate was prepared from mycelium grown 4 days in liquid medium, using a liquid N_2_ grinding procedure for DNA extraction. Two DNA libraries were prepared for sequencing: one a shotgun library and the other a 3-kb paired-end insert library. Each library was sequenced on one picotiter plate using 454 FLX-Titanium chemistry by Roche (5). The >3.1 million 454 pyrosequencing reads were assembled using Roche’s Newbler assembly software. The sequences were assembled into 39 scaffolds for a total genome of 21.9 Mb with a scaffold *N*_50_ measure of 1.5 Mb and a GC content of 47%. Completeness of the assembly was assessed by the presence of 439 (95.9%) of known core eukaryotic genes using CEGMA (6). Genes were predicted using the MAKER version 2.31 annotation pipeline (7) with AUGUSTUS, SNAP, and GENEMARK-ES as gene callers (8–10). The core eukaryotic genes identified by CEGMA (6) and proteins from *Coccidioides immitis* RS were provided as protein evidence to train MAKER, and a total of 7,287 genes were predicted.

### Accession number(s).

This whole-genome shotgun project has been deposited at DDBJ/ENA/GenBank under the accession number MWKM00000000. The version described in this paper is the first version, MWKM01000000.
